# Operationalizing SARS-CoV-2 wastewater monitoring to assess traveler health in Las Vegas, Nevada, USA

**DOI:** 10.1016/j.ijregi.2025.100619

**Published:** 2025-03-05

**Authors:** Casey A. Barber, Ching-Lan Chang, Michael A. Moshi, Shahraiz Akbar, Van Vo, Edwin C. Oh, Daniel Gerrity

**Affiliations:** 1 Applied Research and Development Center, Southern Nevada Water Authority, Las Vegas, NV, USA; 2 Laboratory of Neurogenetics and Precision Medicine, College of Sciences, University of Nevada, Las Vegas, NV, USA; 3 Neuroscience Interdisciplinary Ph.D. program, University of Nevada, Las Vegas, NV, USA; 4 Department of Brain Health, School of Integrated Health Sciences, University of Nevada, Las Vegas, NV, USA; 5 Department of Internal Medicine, Kirk Kerkorian School of Medicine at UNLV, University of Nevada, Las Vegas, NV, USA

**Keywords:** Whole genome sequencing (WGS), Wastewater-based epidemiology (WBE), COVID-19, SARS-CoV-2 variants, Traveler health

## Abstract

**Objectives:**

This study explored expanded traveler- and tourism-focused wastewater monitoring in Las Vegas, Nevada, USA to complement community SARS-CoV-2 surveillance.

**Methods:**

Wastewater samples were collected November 2023 to July 2024 from the largest community-scale wastewater treatment plant in Southern Nevada, USA (N = 112 samples) and two upstream utility access holes (i.e. manholes), isolating an international airport (N = 68 samples) and a commercial area with high-density bars and nightclubs (N = 30-33 samples). Polymerase chain reaction-based methods quantified RNA concentrations of SARS-CoV-2 and pepper mild mottle virus; whole genome sequencing characterized SARS-CoV-2 variants (N = 83 qualifying samples).

**Results:**

SARS-CoV-2 concentrations exhibited concordance between liquids- and solids-based approaches. Similar trends were observed between methods and sampling locations; however, select manhole-level findings suggested potentially divergent COVID-19 infection profiles relative to residents. Whole genome sequencing also demonstrated similarities across sampling locations, although airport samples facilitated the identification of SARS-CoV-2 variants that either failed to spread locally (EG.6, JN.1.11) or preceded detection at the wastewater treatment plant (JN.1.7, KP.3).

**Conclusions:**

These findings offer new insight into the operationalization of broader traveler- and tourism-focused wastewater monitoring, which may capture SARS-CoV-2 concentration spikes and genomic profiles in high-tourism/nightlife areas that community-scale sampling might otherwise miss.

## Highlights

• Travel-focused SARS-CoV-2 wastewater monitoring expanded in Las Vegas, Nevada, USA.
• Liquids- or solids-based methods can be used in site-specific monitoring.
• Select site-specific findings suggest divergent COVID-19 infection profiles.
• Some viral variants isolated to or detected early at the traveler-influenced sites.

## Graphical abstract

**Figure:**
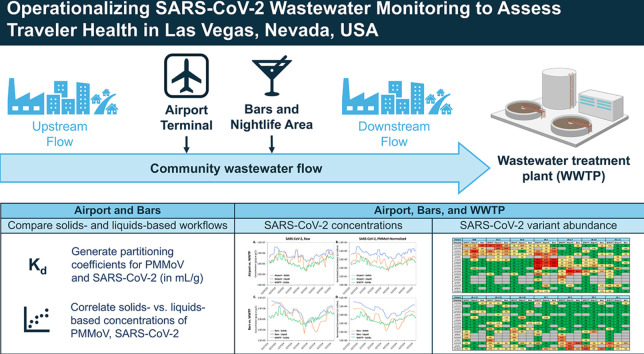

## Introduction

Wastewater-based epidemiology (WBE) leverages the myriad markers of human behavior and disease present in human waste to obtain information about community health status in a robust, non-invasive, and non-identifiable way. Programs like the US Centers for Disease Control and Prevention (CDC) National Wastewater Surveillance System (NWSS) and WastewaterSCAN have expanded the reach of SARS-CoV-2 wastewater monitoring, with much of this work involving surveillance at community-scale wastewater treatment plants (WWTPs). This WWTP-focused approach reflects logistical considerations (e.g*.* ease of sampling) and interest in characterizing COVID-19 incidence across the entirety of the sewershed in a reliable and cost-efficient manner.

Community-level wastewater monitoring, however, may not capture sub-sewershed–level conditions across a geographically large, populous, and/or dynamic sewershed. Equity concerns have also been raised regarding the ability of community-level sampling to assess SARS-CoV-2 burden within high-risk or vulnerable sub-populations [1]. Targeted manhole sampling has shown that communities with higher social vulnerability indexes can exhibit higher wastewater concentrations of SARS-CoV-2 [2], and tailored public health responses may be warranted to better protect these populations. For example, past studies have demonstrated that high-resolution WBE in college dormitories [3] can effectively inform targeted COVID-19 messaging, testing, vaccination, and other interventions.

Travelers represent another sub-population of public health interest, given their potential role in the transmission of novel SARS-CoV-2 variants (among other pathogens) after the post-pandemic return of domestic and international travel. Early travel-focused SARS-CoV-2 wastewater monitoring sampled from aircraft and cruise ships [4]. Wastewater sampling from European airport terminals suggested a high prevalence of COVID-19 among airport passengers and staff in 2022 [5], and airport-specific wastewater concentrations of SARS-CoV-2 RNA have been correlated, with days of lead time, to COVID-19 cases in the surrounding community [6]. Recognizing the potential of these locations to serve as sentinel disease surveillance sites, researchers called for a global aircraft wastewater surveillance network for the early detection, prevention, and mitigation of COVID-19 and future pandemics [7]. In the US, the CDC has conducted airplane and airport wastewater sampling for SARS-CoV-2 variants of concern (VOCs) through its traveler-based genomic surveillance program [8].

Travel- and tourism-focused SARS-CoV-2 wastewater surveillance can extend beyond aircraft and airports. Wastewater surveillance at the Tokyo 2020 Olympic and Paralympic Village [9], for example, demonstrated how WBE can be applied at international events and mass gatherings. Cities that are international tourism destinations can also serve as sentinel SARS-CoV-2 surveillance sites, as Sangsanont et al. [10] demonstrated in their study of Bangkok and Phuket, Thailand. Las Vegas, Nevada is another international tourism hub (welcoming over 40.8 million visitors in 2023 [11]) where community-wide SARS-CoV-2 wastewater monitoring has been conducted since 2020. Recent manhole sampling [12] demonstrated that visitors to the casino-resort corridor known as the Las Vegas Strip have a considerable impact on wastewater SARS-CoV-2 loadings. In fact, analysis of wastewater samples from the Las Vegas Strip yielded the first confirmed Omicron VOC detection in Southern Nevada—preceding the first clinical case by approximately 1 week [12]. These examples highlight how strategic wastewater sampling might offer a novel way to monitor traveler health.

To further explore operationalization of wastewater monitoring for traveler health surveillance, this study sampled from three sites in a tourism-influenced area in Las Vegas: the international airport, a cluster of bars/nightclubs near the Las Vegas Strip, and the downstream WWTP. Wastewater targets of interest included the RNA of SARS-CoV-2 and pepper mild mottle virus (PMMoV) (a human fecal indicator). Specifically, this study characterized solid vs liquid partitioning of the RNA targets to better understand the nuances of the solids- and liquids-based methods used by two major contract laboratories. This study also monitored trends in absolute and PMMoV-normalized SARS-CoV-2 concentrations, as well as SARS-CoV-2 variant abundance, to facilitate comparisons between the two tourism-influenced sites and the community-scale WWTP. Collectively, this information provides important guidance to wastewater surveillance decision-makers regarding the adoption of traveler-focused wastewater monitoring and the implications of the different methods used by wastewater surveillance programs.

## Methods

### Wastewater sampling locations

#### Community-scale wastewater treatment plant

Composite influent wastewater samples (24-hour, flow-weighted) were collected using an autosampler (Hach AS950; Loveland, CO, USA) at the largest WWTP in Southern Nevada (shown in Supplementary Figure S1). This facility treats approximately 100 million gallons per day (mgd) of wastewater, serving approximately 1 million residents and 700,000 weekly visitors to the Las Vegas Strip. This sewershed includes the University of Nevada, Las Vegas (UNLV) (approximately 30,000 students) and a large international airport that welcomed 57.6 million airport travelers in 2023 [11]. As part of the national WastewaterSCAN program [13], samples were collected three times per week (Monday/Wednesday/Friday) between November 17, 2023 and July 31, 2024 (N = 112 samples) and shipped on ice overnight to a contract laboratory (Verily Life Sciences, South San Francisco, CA, USA) for quantification of SARS-CoV-2 and PMMoV RNA. A subset of these samples was transported to the UNLV laboratory for amplicon-based whole genome sequencing (WGS).

#### International airport

Sampling was conducted twice per week between November 17, 2023 and July 31, 2024 (N = 68 samples each for Biobot and Verily) at a manhole serving the international airport (Supplementary Figures S1, S2), hereafter the “airport” sample. This manhole isolated sewage flows from Terminal 1 (domestic flights only) but represented a combination of sources, including aircraft (i.e. triturator discharge), air travelers, and airport staff and guests. Approximately 7 L of wastewater were collected in a 10-L carboy housed within an autosampler (Hach AS950; Loveland, CO, USA) positioned on a bed of ice. Starting at 2:00 pm every Monday and Thursday, the autosampler collected 50 ml of sewage every 10 minutes for 24 hours. Samples were retrieved at ∼2:00 pm every Tuesday and Friday, transported to the UNLV laboratory for short-term storage and WGS processing, and aliquots were shipped on ice to the two contract laboratories: Verily Life Sciences and Biobot Analytics (Cambridge, MA, USA).

#### Cluster of bars/nightclubs near the Las Vegas Strip

Sampling was conducted once per week between December 2, 2023 and July 27, 2024 (N = 30 samples for Biobot and 33 samples for Verily) at a manhole that isolated sewage flows primarily from a commercial development consisting of high-density bars and nightclubs (Supplementary Figures S1, S3), hereafter the “bars” sample. Approximately 8 L of sewage were collected in a 10-L carboy housed within an autosampler (Hach AS950; Loveland, CO, USA) positioned on a bed of ice. The autosampler collected 50 ml of sewage every 5 minutes between 5:00 pm Friday night and 6:00 am Saturday morning. This sampling interval targeted peak customer capacity of the bars/nightclubs late at night while minimizing possible confounding contributions from other small businesses in the immediate vicinity. Samples were retrieved every Saturday morning, transported to the UNLV laboratory for short-term storage and WGS processing, and aliquots were shipped on ice to Verily Life Sciences and Biobot Analytics.

### Sample processing and analysis

#### Liquids- and solids-based quantification of RNA targets (contract laboratories)

Biobot Analytics analyzed for liquid-phase targets, whereas Verily Life Sciences focused on solid-phase targets. Because influent wastewater is predominantly liquid in nature (on the order of 0.01% solids), most of the overall SARS-CoV-2 and PMMoV load is expected to be present in the liquid phase. However, multiple studies [14,15] have demonstrated that viral targets can be concentrated >100-1000–fold in the solid fraction on an equivalent mass basis (e.g. 1 ml of liquid wastewater vs 1 g of wastewater solids). Notably, the analysis of wastewater solids, whether isolated via centrifugation or collected directly from WWTP clarifiers, does not require labor-intensive concentration of liquid wastewater. Details of the liquids-based methods (Biobot Analytics) [16] and the solids-based methods (Verily Life Sciences) [15,17,18] for sample preparation and polymerase chain reaction–based RNA target quantification are included in the supplementary material (Supplementary Text S1). PMMoV-normalized concentrations represent the ratio of the SARS-CoV-2 concentration to the PMMoV concentration, multiplied by 1 million for scaling purposes.

#### WGS approach (University of Nevada, Las Vegas laboratory)

A subset of the wastewater samples (N = 177) was processed and analyzed by amplicon-based WGS using the CleanPlex SARS-CoV-2 FLEX Panel from Paragon Genomics (Fremont, CA, USA), as described previously [19]. All libraries were sequenced on an Illumina NextSeq 1000 instrument using P1 300-cycle flow cells. Raw sequencing data underwent trimming and filtering using fastp v0.23.4 [20] to remove Illumina adapters, low-quality reads (quality score <20), and polyG tails. The processed reads were then aligned to the SARS-CoV-2 reference genome (NC_045512.2) using BWA mem v0.7.17-r1188 [21]. SAMtools v1.15.1 [22] was used to sort and index the aligned reads. Using fgbio TrimPrimers v2.1.0 with hard clipping enabled, tiled amplicon primers were removed from the sorted reads. Variant calling was performed using iVar v1.3.2 [23]. Genome coverage metrics were calculated from SAMtools depth output (Supplementary Tables S1-S3). For quality control, samples were required to achieve ≥70% genome coverage at 50× depth or greater. The data passing these quality thresholds were analyzed using Freyja v1.4.7 to estimate SARS-CoV-2 lineage abundances within the wastewater samples. This analysis used a barcode matrix derived from the UShER global phylogenetic tree, incorporating lineages specific to this study. We report variants showing a relative abundance of at least 3% in one or more samples at any point during the study. Using this threshold, this study focused on changes in major variants at each sampling location over time rather than attempting to identify rare variants or cryptic lineages. The percentages reported later represent the average relative abundance across all samples meeting the established quality control criteria in each 2-week sampling period.

### Data analysis

This study assessed solid vs liquid partitioning of the RNA targets (SARS-CoV-2 and PMMoV) in the context of a partitioning coefficient, K_d_ (ml/g) [14,15], which was calculated as the ratio of the target concentration in the solid fraction, C_S_ (in gene copies per gram, gc/g), and the target concentration in the liquid fraction, C_L_ (in gene copies per milliliter, gc/ml). K_d_ values were not calculated for this study's community-scale WWTP samples because there was no corresponding liquids-based concentration. Wilcoxon signed-rank and Kruskal–Wallis tests were used to compare K_d_ values as a function of virus and sampling location, Spearman correlations and linear regressions were used to assess relationships between solids- and liquids-based RNA concentrations and across the three sampling locations, and three-point moving averages were used to illustrate concentration trends.

## Results and discussion

### SARS-CoV-2 and PMMoV partitioning

The median K_d_ values for PMMoV and SARS-CoV-2 were significantly different (Wilcoxon signed-rank *P* <0.01) than the median K_d_ values reported in the literature [14,15] (Table 1; norovirus GII K_d_ values [24] included for comparison), although they were within the same order of magnitude. There are likely many factors that contribute to differences in K_d_ values for a given virus, including wastewater temperature and ionic strength, so some differences between studies are not entirely unexpected. The fact that the liquids and solids data used in calculating K_d_ were generated by two different laboratories, as in the study by Kim et al. [14], may have also contributed experimental/methodological variability.

**Table 1:** Summary of virus partitioning coefficients (K_d_, ml/g) from the literature and the pooled data set from the current study (i.e. all airport and bars samples). Norovirus genotype II (NoV GII) is also included for comparison.

	Partitioning coefficients (K_d_, in ml/g) by virus					
	Pepper mild mottle virus			SARS-CoV-2		NoV GII
	Kim et al. [14]	Boehm et al. [15]	Current study	Kim et al. [14]	Current Study	Boehm et al. [24]
Min.	4×10^2^	1.6×10^3^	2.1×10^2^	-	6.7×10^1^	2.1×10^3^
25th	-	-	2.0×10^3^	3×10^2^	1.9×10^2^	-
Median	6×10^3^	2.4×10^3^	3.1×10^3^	9×10^2^	3.1×10^2^	2.5×10^3^
75th	-	-	5.4×10^3^	4×10^3^	8.5×10^2^	-
Max.	3×10^5^	3.3×10^3^	6.2×10^4^	-	7.2×10^3^	3.9×10^3^

**Figure 1:**
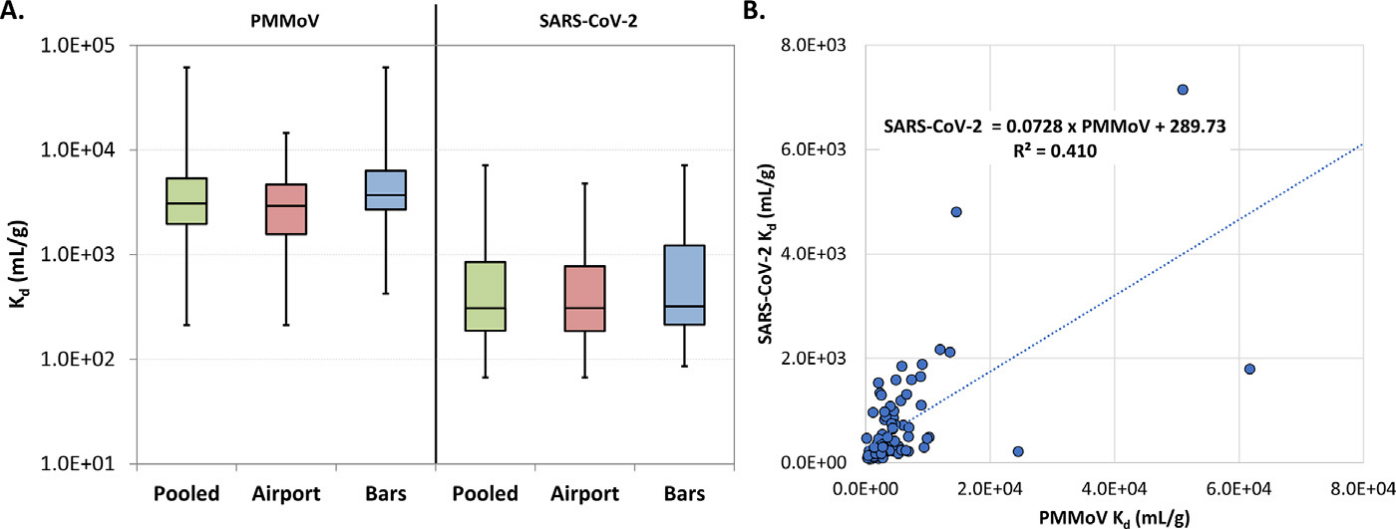
(a) Distribution of partitioning coefficients (K_d_, ml/g) for PMMoV and SARS-CoV-2. Boxes represent the 25th, 50th, and 75th percentiles, and whiskers indicate minima and maxima. The “pooled” values (green boxes) represent all values from the airport and bars samples. (b) Linear relationship between K_d_ values for SARS-CoV-2 vs PMMoV. PMMoV, pepper mild mottle virus.

Within this study (Figure 1a), SARS-CoV-2 K_d_ values were similar across manhole sampling locations (bars vs airport Kruskal–Wallis *P* = 0.34), whereas the PMMoV K_d_ values were slightly higher in the bars samples than the airport samples (Kruskal–Wallis *P* = 0.02). Between the two viruses, the median K_d_ value was approximately 10-fold higher for PMMoV than SARS-CoV-2, regardless of sampling location (Figure 1a). Higher K_d_ values indicate that PMMoV generally partitioned to solids (or was solids-associated) to a greater extent than SARS-CoV-2 in these samples. However, the moderate correlation between the pooled K_d_ values of the two viruses (Spearman r = 0.65; *P* <0.0001; linear R^2^ = 0.410; Figure 1b) suggests that, despite wide ranges in absolute K_d_ values for each virus, relative partitioning on a sample-specific basis was somewhat stable. Importantly, this implies that normalizing SARS-CoV-2 to PMMoV may help correct for operational/methodological variability (e.g*.* increases in solid partitioning) not driven by epidemiologic factors. PMMoV normalization has been applied widely in SARS-CoV-2 WBE [16,17,25], although a variety of normalization factors have been explored [25]. Although wastewater concentrations may vary by season, region, dietary habits, and urbanization [25], PMMoV is an abundant human fecal indicator expected to be consistent at large WWTPs. Here, these data provide some justification for its use as a normalization factor even at smaller spatial scales, such as the two manhole locations.

### Solids-based vs liquids-based quantification of RNA concentrations

Both solids- and liquids-based methods can be highly sensitive options for SARS-CoV-2 WBE applications. In this study, PMMoV was detected in 100% of the WWTP, airport, and bars samples, highlighting its reliability as a human fecal indicator. For the solids- and liquids-based methods, SARS-CoV-2 was detected in 96% and 100% of the airport samples and 88% and 93% of the bars samples, respectively. Coincidentally, solids- and liquids-based concentrations may appear similar in magnitude because of the 100-1000–fold concentration factor associated with solid partitioning (for solids-based concentrations) being offset by the 1000-fold unit conversion between milliliters and liters (for liquids-based concentrations). Although it is inappropriate to compare the concentrations on an absolute basis because of differential partitioning, reporting absolute concentrations here (Supplementary Table S4) facilitates comparisons with other data sets in the literature generated using similar methods, or between two time points within the same data set to compare changes over time.

**Figure 2:**
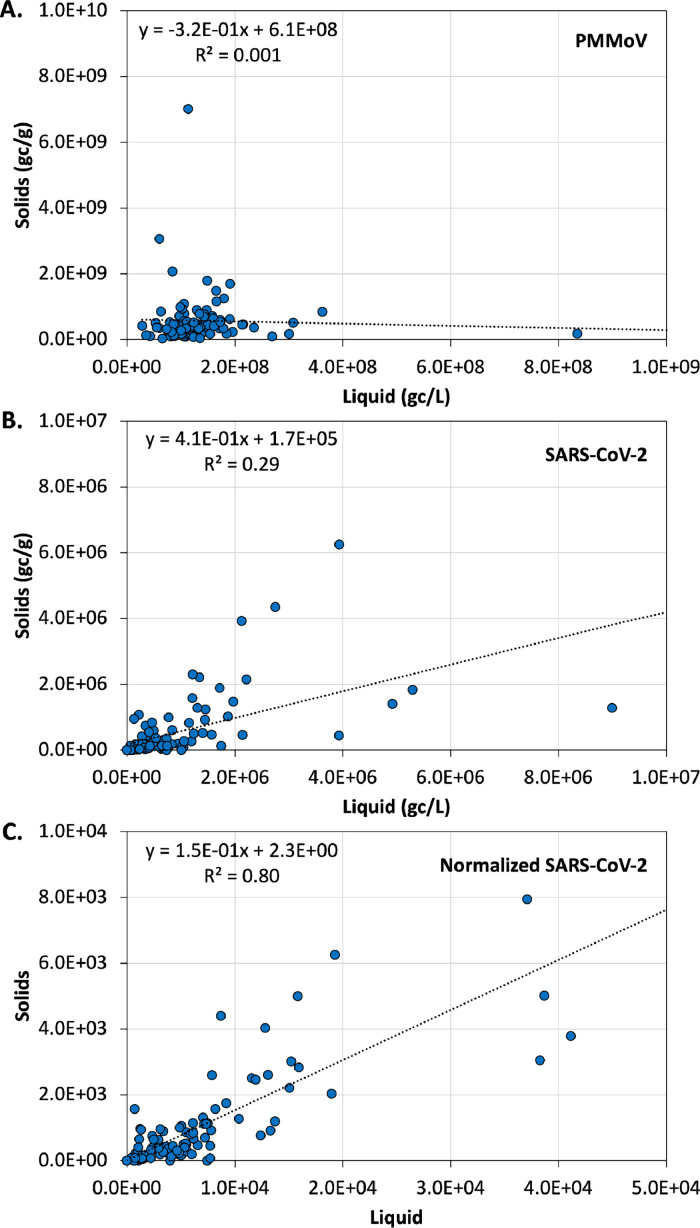
Linear relationships between solids- and liquids-based (a) PMMoV, (b) SARS-CoV-2, and (c) PMMoV-normalized SARS-CoV-2 concentrations (multiplied by 1 million for scaling purposes). The plots include the pooled data set for all airport and bars samples. PMMoV, pepper mild mottle virus.

Rather than absolute concentrations, correlations and linear relationships offer more appropriate comparisons of solids- and liquids-based methods. For PMMoV (which was highly abundant at ∼10^8^ gc/g or gc/ml), there was no evidence of a linear relationship (R^2^ = 0.0013) between the solids- and liquids-based data (Figure 2a), possibly influenced by outliers in both approaches; however, there was some indication of a Spearman correlation (r = 0.19; *P* = 0.06). SARS-CoV-2 solids- and liquids-based concentrations demonstrated stronger relationships (Spearman r = 0.68; *P* <0.0001; linear R^2^ = 0.29) but still exhibited considerable sample-specific divergence (Figure 2b). Finally, PMMoV-normalized SARS-CoV-2 yielded the strongest correlations between the solids- and liquids-based data (Spearman r = 0.75; *P* <0.0001; linear R^2^ = 0.80) (Figure 2c), presumably because normalization serves as an internal “correction” for sample-specific variability in recovery, human contributions (e.g*.* fecal loads), partitioning, etc. This finding further supports PMMoV normalization in current national wastewater surveillance programs.

### SARS-CoV-2 longitudinal concentration profiles across sampling locations

**Figure 3:**
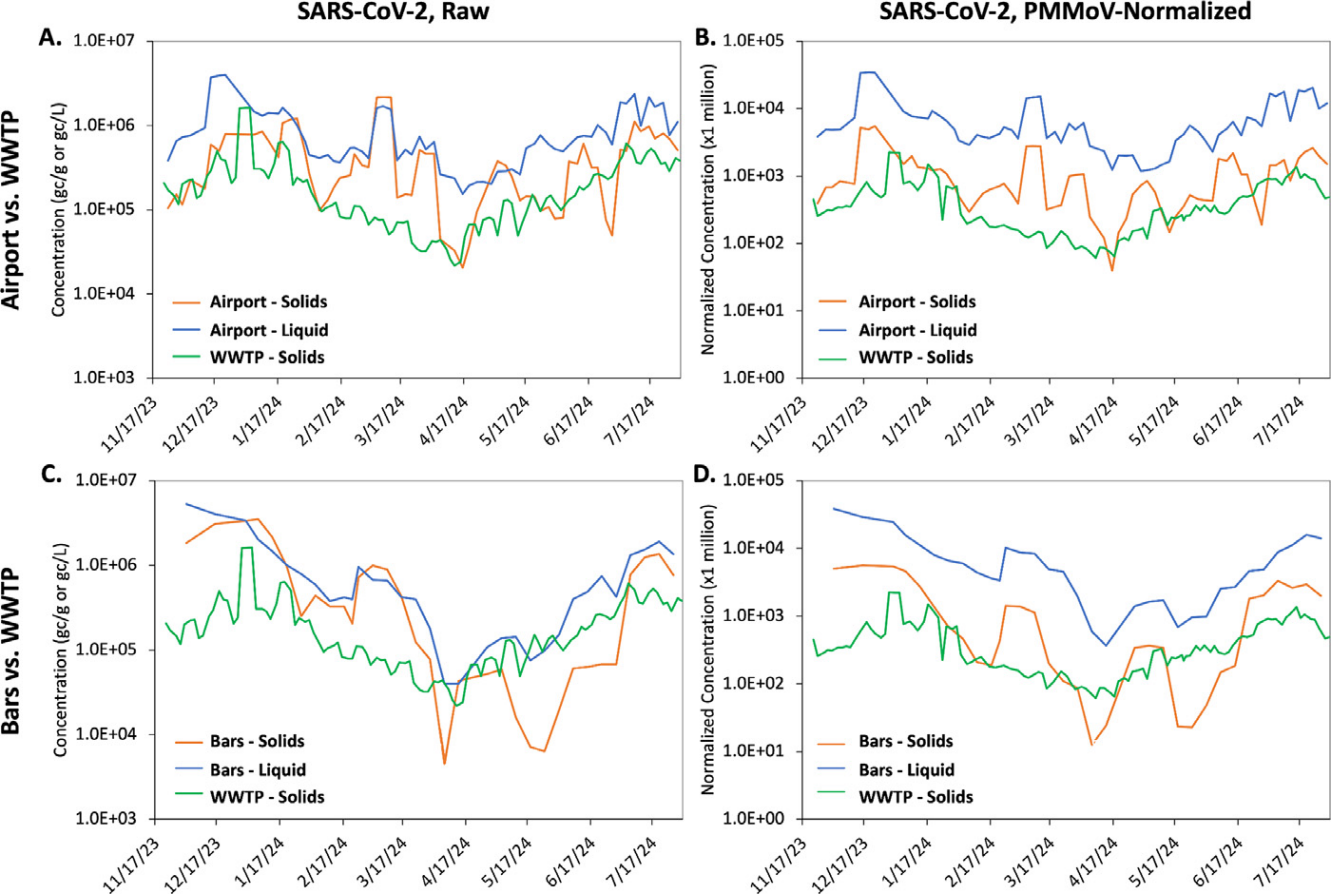
Longitudinal raw and PMMoV-normalized SARS-CoV-2 concentration profiles highlighting the (a, b) airport and (c, d) bars compared with the WWTP (shown in green). (b, d) PMMoV-normalized SARS-CoV-2 concentrations were calculated as the ratio of SARS-CoV-2 to PMMoV, multiplied by 1 million for scaling purposes. Each line represents a three-point moving average of the underlying raw data. Solids-based data (orange and green) are reported in gc/g, and liquids-based data (blue) are reported in gc/l, whereas the normalized concentrations are unitless. PMMoV, pepper mild mottle virus; WWTP, wastewater treatment plant.

Three-point moving averages of the raw and PMMoV-normalized SARS-CoV-2 concentrations over the course of the study are shown in Figure 3. The higher-resolution airport/bars manhole data generally tracked the community-scale WWTP data with moderate correlations for the raw (Spearman r = 0.49-0.76; *P* <0.01) and PMMoV-normalized (Spearman r = 0.45-0.65; *P* <0.01) SARS-CoV-2 concentrations (Supplementary Table S5). To reconcile the different sampling frequencies/days across the three locations, weekly (Sunday-Saturday) mean concentrations were used to evaluate these correlations. All concentrations peaked in winter 2023, declined in early spring 2024, and then increased in late spring/early summer 2024. This seasonal pattern is consistent with community-scale wastewater data from previous years [12]. There were also moderate correlations (Spearman r = 0.51-0.68; *P* <0.01) between the solids- and liquids-based concentrations at both of the manhole sites over time, suggesting that either method can capture trends in transmission (Supplementary Table S5). Importantly, the similarities across the three locations (airport, bars, and WWTP) suggest that visitors/tourists were generally exhibiting SARS-CoV-2 infection profiles similar to the surrounding community, although contributions from local residents are also possible at these traveler-influenced manhole locations. This study did not isolate residential flows for comparison, which would be important for confirming whether a unique signal at a traveler-influenced location was truly originating from travelers.

Sampling from the traveler-influenced manholes revealed unique changes in SARS-CoV-2 wastewater concentrations that were not observed at the downstream WWTP. The clearest example of this occurred in early March 2024, when concentrations at both manhole locations rose sharply to a peak not seen in the corresponding WWTP data (which was declining at the time). Similarly, a rapid drop in concentration (i.e*.* a trough) was observed at the bars location in late May 2024 but not at the WWTP. The fact that similar peaks/troughs were observed for the split samples across the two laboratories/methods suggests that these were true changes in concentration at the manhole locations. Discrepancies between upstream and community-scale sampling locations may sometimes be an artifact of dilution at the WWTP (i.e. a smoothing effect); however, they may also be indicative of localized changes in disease transmission, some of which may warrant targeted responses. This highlights the potential public health value of upstream wastewater monitoring and, again, the importance of “local” sentinel sites for comparing resident-dominated locations against traveler-influenced locations.

In contrast with the WWTP samples (which all contained quantifiable concentrations of the RNA targets), there were several SARS-CoV-2 non-detects at the manhole sites from late March through June 2024. Most of these non-detects were also consistent between the two laboratories/methods, supporting the conclusion that they were true non-detects. It is important to note that the manhole samples represent fewer contributing individuals than the WWTP, and non-detects may be more likely during low-prevalence periods among smaller contributing populations. It is also possible that manholes in concentrated tourist areas can capture true population-specific differences (e.g*.* temporarily lower COVID-19 incidence among travelers relative to locals); however, further elucidation of exclusively local/residential flows would allow further distinction between local vs traveler contributions.

Discrepancies between the manhole samples and the community-scale WWTP might also reflect different health profiles of travelers after the pandemic [26], particularly if individuals with symptomatic COVID-19 or more COVID-19–related risk factors choose to forgo travel. These differences also highlight the importance of diversity/redundancy in wastewater sampling locations for traveler health surveillance programs (i.e*.* not only airports and aircraft) because some passengers report never defecating on flights (>50% for short-haul and 20-30% for long-haul flights) or at departure (20-30%) or arrival airports (30-40%) [27].

### WGS of SARS-CoV-2

**Figure 4:**
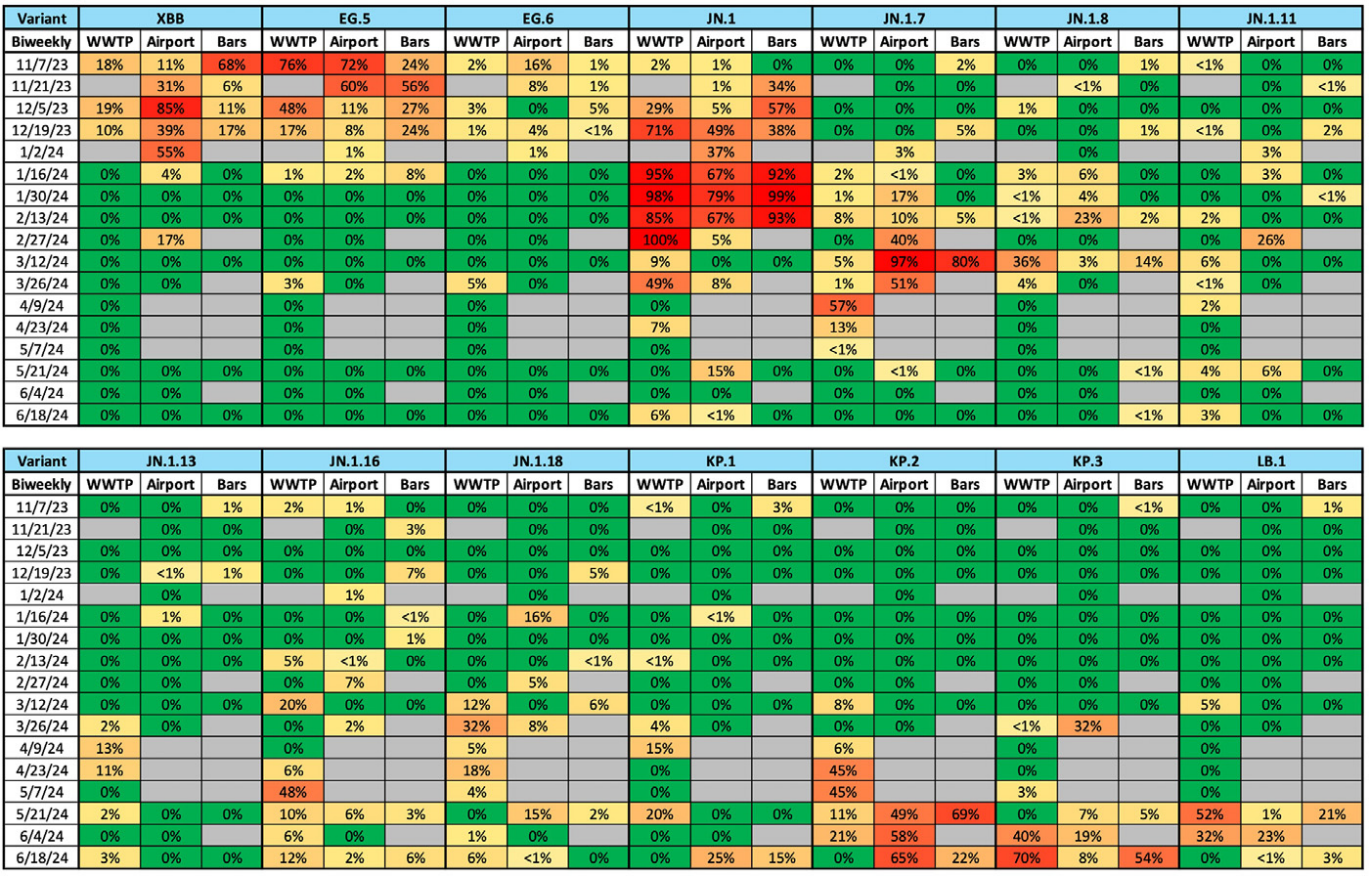
Relative abundance of SARS-CoV-2 variants in samples collected from the community-scale WWTP, the airport manhole, and the bars manhole. Variants are included if they accounted for at least 3% relative abundance in one or more samples. Percentages represent average relative abundance across all samples meeting established quality control criteria (minimum 70% coverage at 50× depth) in each 2-week sampling period. Gray cells indicate periods for which no samples met the established quality control criteria for a given location, and green cells indicate true non-detects for that variant. WWTP, wastewater treatment plant.

After applying rigorous quality control measures, 83 of 177 samples analyzed by WGS met established sequencing metrics to undergo comprehensive variant analysis (Supplementary Tables S1-S3). The results revealed distinctive patterns in variant detection, abundance, and distribution over time (Figure 4). Of the 14 SARS-CoV-2 variants exceeding 3% relative abundance during this timeframe, most were identified concurrently across the three locations, including XBB and EG.5 (November/December 2023), JN.1 (December 2023-February 2024), and the various FLiRT variants plus LB.1 (March-June 2024). Local VOC reporting for clinical samples changed in October 2023; however, the wastewater findings here mirror the national predominance of XBB descendants (summer/fall 2023) and JN.1 (late 2023 and spring/summer 2024) in clinical samples [28]. These variant similarities between local-dominated wastewater, traveler-influenced wastewater, and national clinical data may reflect the fact that Terminal 1 serves domestic flights only and that Las Vegas visitors are primarily US residents, with only 12% of visitors originating from international locations in 2023 [29]. However, the airport samples also facilitated identification of variants that either (1) failed to spread from travelers to the local community (EG.6) or (2) foreshadowed weeks-later detection at the downstream WWTP (JN.1.7 and KP.3). These unique detections also underscore how upstream sampling can facilitate detection of signals that might otherwise be diluted out by the larger flows at a community-scale WWTP.

## Conclusion

Informing the operationalization of future traveler-focused wastewater monitoring efforts, this study identified alignment between the SARS-CoV-2 concentrations from two commercial laboratories applying different (solids- vs liquids-based) analytical methods to samples from traveler-influenced manholes and a downstream WWTP. These results support PMMoV normalization and the use of either solids- or liquids-based approaches in future similar work. Furthermore, although longitudinal monitoring suggests some similarities between the manholes and downstream WWTP, there were periods when samples from traveler-influenced sites (airport/bars) diverged from the downstream WWTP. These results reaffirm the notion that higher-resolution wastewater surveillance has the capacity to detect changes in pathogen occurrence within specific sub-populations that might otherwise go undetected at community-scale WWTPs. More broadly, this study demonstrates the feasibility of longitudinal, traveler-focused wastewater monitoring in and beyond airport settings while also highlighting important limitations for future efforts, including the confounding effects of dilution and the mixing of local and visitor contributions at traveler-influenced sites. Future efforts should consider including local-dominated sentinel sites to facilitate direct comparisons.

Timely detections of novel/emerging public health threats in international travel destinations have potentially vast implications for global health and the global economy. Future work may elucidate optimal high-resolution wastewater sampling strategies but must still protect individual privacy, prevent data misuse, and avoid unintended consequences for vulnerable communities. Innovative approaches to higher-resolution wastewater monitoring present opportunities for previously unrealized potential in public health surveillance, building on recent advances with wastewater surveillance of other pathogens, such as influenza [30].

## Declarations of competing interest

Edwin Oh reports financial support was provided by Centers for Disease Control and Prevention. One of the contract laboratories mentioned in this study (Verily Life Sciences) provided wastewater analyses to an external collaborator (Clark County Water Reclamation District) free of charge. These analyses were unrelated to the current study and did not impact the design, methods, results, or conclusions of this study. The analyses conducted by Biobot Analytics and Verily Life Sciences in the current study were paid for through contractual agreements with the aforementioned grant funding. All other authors declare that they have no known competing financial interests or personal relationships that could have appeared to influence the work reported in this paper.

## Appendix Supplementary materials

Image, application 1

## Footnotes

Supplementary material associated with this article can be found, in the online version, at doi:10.1016/j.ijregi.2025.100619.
