# Supplementary material for: Operationalizing SARS-CoV-2 wastewater monitoring to assess traveler health in Las Vegas, Nevada, USA
Source: IJID Reg. 2025 Mar 5;15:100619. doi: 10.1016/j.ijregi.2025.100619 (PMC11964744; doi:10.1016/j.ijregi.2025.100619)
Supplement: Supplementary file 1 [file mmc1.pdf]

Supplementary Material

Title: Operationalizing SARS-CoV-2 Wastewater Monitoring to Assess Traveler Health in Las Vegas, Nevada, USA

Authors: Casey A. Barber, PhD [a,1]; Ching-Lan Chang, BSc [b,c,1]; Michael A. Moshi, BSc [b,c]; Shahraiz Akbar, BA [b]; Van Vo, PhD [b]; Edwin C. Oh, PhD [b,c,d,e], Daniel Gerrity, PhD [a*]

[a] Applied Research and Development Center

Southern Nevada Water Authority, P.O. Box 99954, Las Vegas NV, 89193, USA.

[b] Laboratory of Neurogenetics and Precision Medicine, College of Sciences

[c] Neuroscience Interdisciplinary Ph.D. program

[d] Department of Brain Health, School of Integrated Health Sciences

[e] Department of Internal Medicine, Kirk Kerkorian School of Medicine at UNLV

University of Nevada, Las Vegas (UNLV), 4505 S. Maryland Pkwy, Las Vegas, NV 89154, USA

[1] These first authors contributed equally to this article.

[*]**To whom correspondence should be addressed:** Daniel Gerrity (daniel.gerrity@snwa.com)

Supplementary Figure S1. Sewershed delineations in Southern Nevada. The green dots represent the three sampling locations for this study: the community-scale wastewater treatment plant (WWTP), the "Airport" manhole, and the "Bars" manhole. The Las Vegas Strip is also located within this sewershed, immediately west/upstream of the manhole sampling locations.

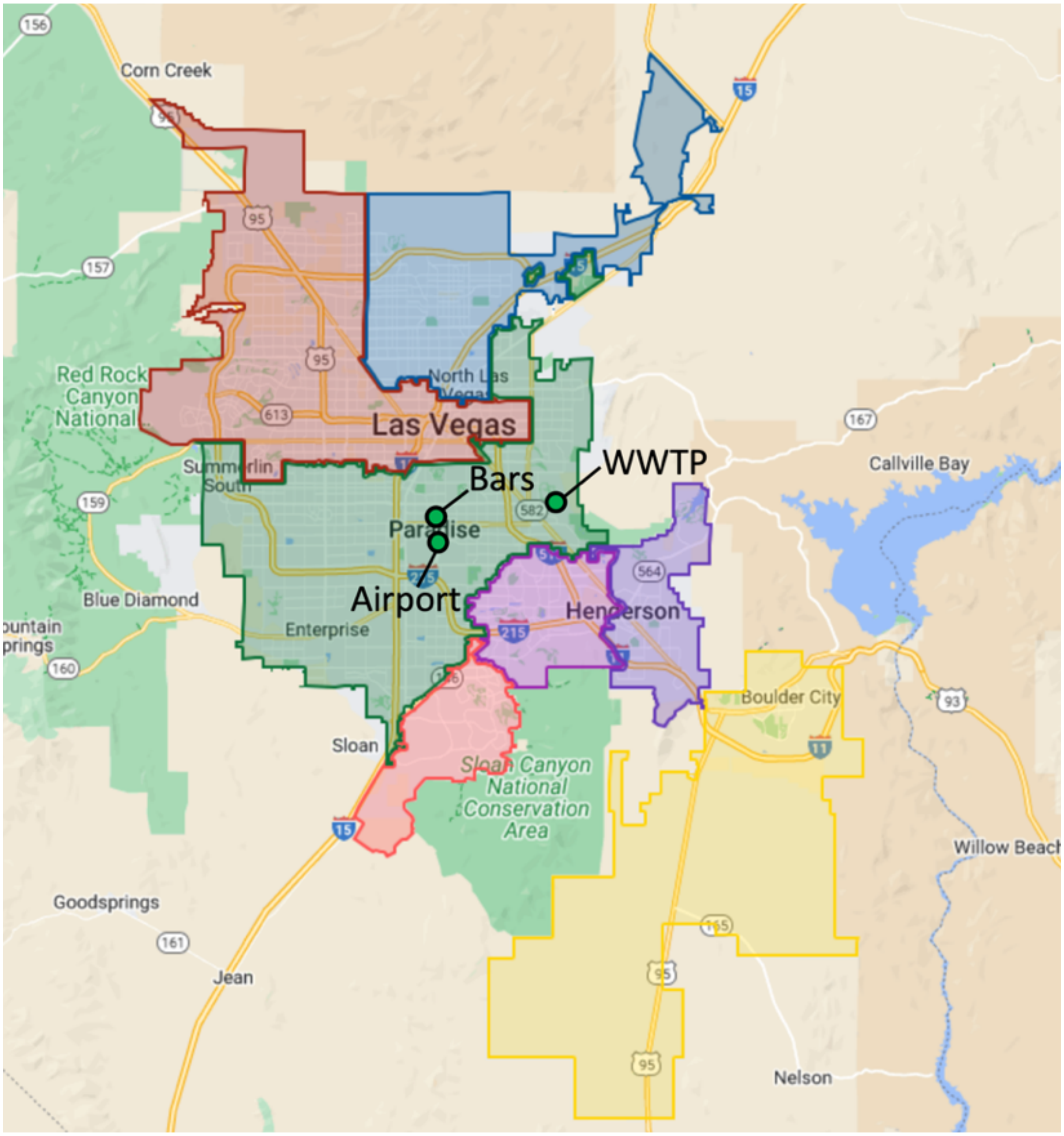

Supplementary Figure S2. Satellite imagery of (**left**) the airport and (**right**) inset of the "Airport" sampling location (manhole denoted by red circle). The sewer line (green) originates at the airport. **Source:** Google Maps and AJ Rodrigues (Clark County Water Reclamation District).

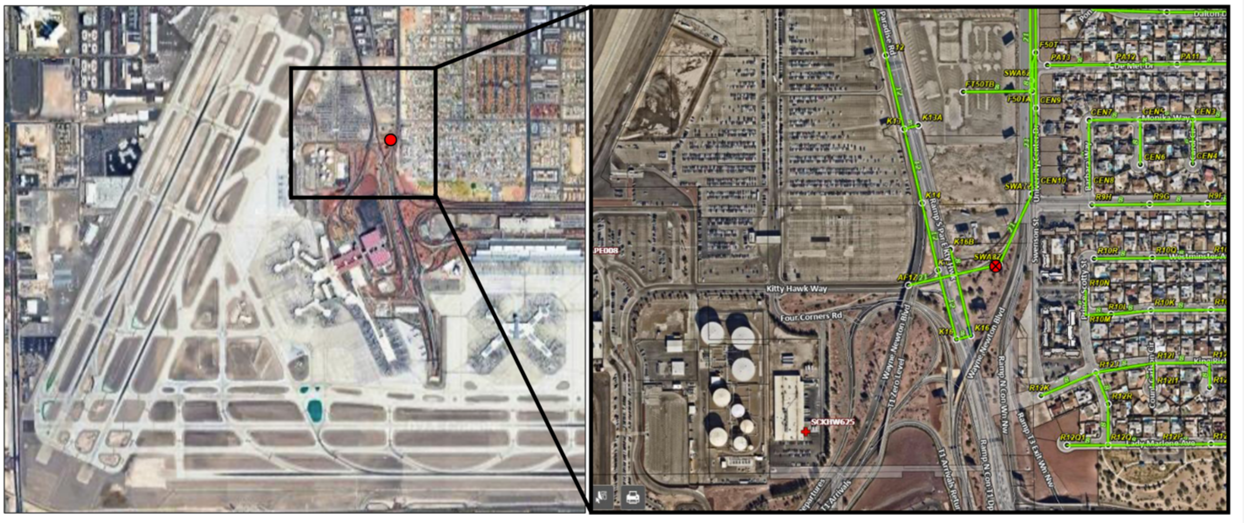

Supplementary Figure S3. Satellite imagery of the “Bars” sampling location. The bars/nightclubs are denoted by the black rectangle, and the manhole is denoted by the red circle. The sewer line (green) originates on the left side of the map. **Source:** Google Maps and AJ Rodrigues (Clark County Water Reclamation District).

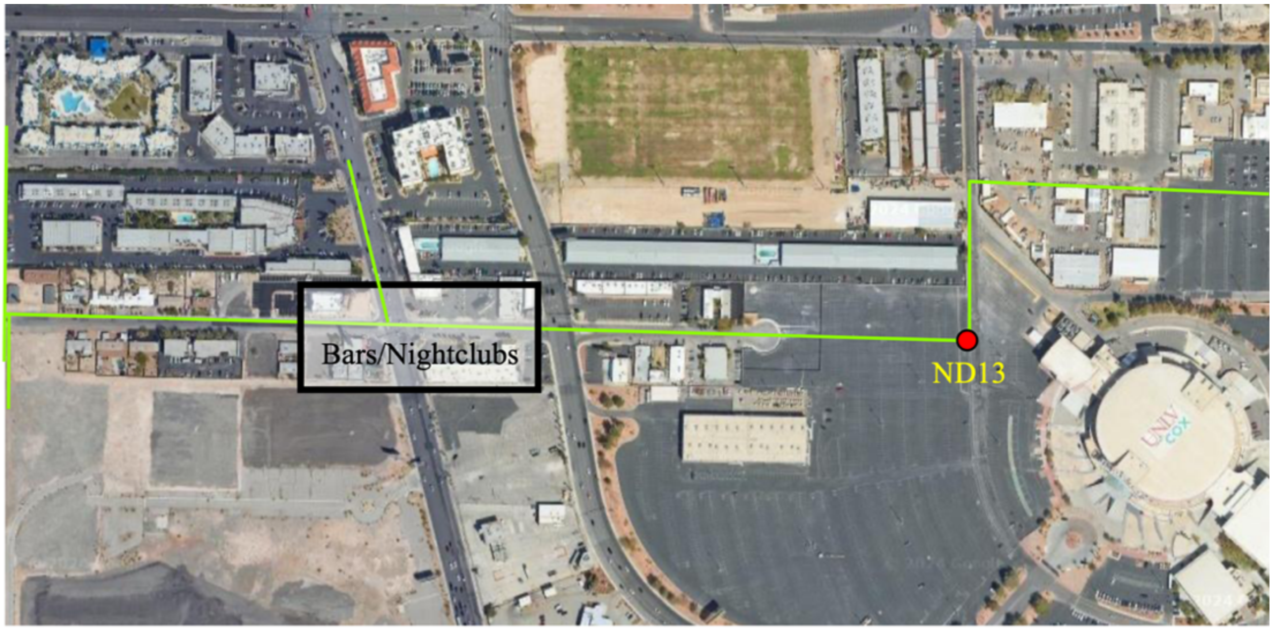

Supplementary Text S1. Sample preparation and target quantification methods used by the contract laboratories.

Liquids-based Approach (Biobot Analytics)

Biobot Analytics processed the wastewater samples using vacuum filtration (0.2 μm, Steriflip, EMD Millipore, Burlington, MA) to remove large particulates, followed by centrifugal ultrafiltration (Amicon Ultra-15, Millipore) to achieve ~100-fold concentration. Nucleic acids were extracted using AVL Buffer (Qiagen, Hilden, Germany) and RNeasy Mini columns or RNeasy 96 cassettes (Qiagen), and nucleic acids were eluted in 75 μL of nuclease-free water. 3 μL of the nucleic acid eluate were then analyzed using one-step reverse transcription quantitative polymerase chain reaction (RT-qPCR) in triplicate reactions on a CFX96 qPCR instrument (Bio-Rad, Hercules, CA). The RT-qPCR assays targeted the N1 and N2 genes of SARS-CoV-2, along with PMMoV as an indicator of fecal load. Ct values for the qPCR assays were converted to gene copies (gc) using synthetic RNA (SARS-CoV-2) or gBlock Gene Fragment (PMMoV) standard curves and then divided by equivalent sample volumes (ESVs) to calculate concentrations in units of gc/L. Reported SARS-CoV-2 concentrations represented the overall average of all N1 and N2 replicates for a given sample. Additional details, including primer and probe sequences, are provided in Duvallet et al.[1]

Solids-based Approach (Verily Life Sciences)

Although only the WWTP samples were analyzed as part of the official WastewaterSCAN program, the WWTP samples and the manhole samples were all analyzed by Verily using the same approach. Solids were first isolated by centrifuging 50-mL wastewater

samples at 24,000×g for 30 min at 4ºC. The supernatant was discarded and approximately 0.75 g of solids were transferred to a new conical tube for homogenization and nucleic acid extraction; a portion of the remaining solids were aliquoted for determination of percent solids content. DNA/RNA shield was added to the solids along with 5-10 grinding balls (5/32 in.; OPS Diagnostics), and the samples were processed using an automated tissue homogenizer and cell lyser (Geno/Grinder 2010; Spex SamplePrep, Cole-Parmer) at 1,000 rpm for 2 min. Samples were then centrifuged at 5,250×g for 5 min. Nucleic acids were extracted from 300 µL of this homogenate using a Chemagic 360 automated system (Perkin Elmer Revvity) and a Chemagic Viral DNA/RNA 300 Kit H96, followed by inhibitor removal with the Zymo OneStep-96 PCR Inhibitor Removal Kit. 5.5 µL of template were analyzed in 10 replicate wells for SARS-CoV-2 and PMMoV using one-step RT-PCR on a droplet digital PCR (ddPCR) platform. Droplets were generated with an AutoDG Automated Droplet Generator, PCR was performed with a Mastercycler Pro, and droplets were analyzed with a QX200 Droplet Reader (Bio-Rad). Quantities determined from the ddPCR assays were adjusted for dilution (when applicable) and ESV to report concentrations as gc/g (dry weight). Additional sample processing details are available in Wolfe et al.,[2] and ddPCR assay details have been published previously.[3,4]

Supplementary Text S1 References

1. Duvallet C, Wu F, McElroy KA, et al. Nationwide Trends in COVID-19 Cases and SARS-CoV-2 RNA Wastewater Concentrations in the United States. *ACS ES&T Water*. 2022;2(11):1899-1909. doi:10.1021/acsestwater.1c00434
2. Wolfe MK, Topol A, Knudson A, et al. High-Frequency, High-Throughput Quantification of SARS-CoV-2 RNA in Wastewater Settled Solids at Eight Publicly Owned Treatment Works in Northern California Shows Strong Association with COVID-19 Incidence. *mSystems*. 2021;6(5):e00829-21. doi:10.1128/mSystems.00829-21
3. Boehm AB, Wolfe MK, Wigginton KR, et al. Human viral nucleic acids concentrations in wastewater solids from Central and Coastal California USA. *Sci Data*. 2023;10(1):396. doi:10.1038/s41597-023-02297-7
4. Boehm AB, Wolfe MK, Bidwell AL, et al. Human pathogen nucleic acids in wastewater solids from 191 wastewater treatment plants in the United States. *Sci Data*. 2024;11(1):1141. doi:10.1038/s41597-024-03969-8

Supplementary Table S1. Whole genome sequencing (WGS) metrics for the community-scale wastewater treatment plant (WWTP) samples. For quality control, samples were required to achieve ≥70% genome coverage at 50x depth or greater.

Location	Date	Reads	Coveredby1x	Coveredby10x	Coveredby30x	Coveredby50x	Coveredby100x
WWTP	11/08/23	1,451,709	99.62%	99.28%	98.43%	97.68%	95.15%
WWTP	11/10/23	1,298,111	99.75%	99.12%	97.89%	96.18%	92.56%
WWTP	12/06/23	317,798	99.85%	96.34%	86.93%	78.89%	66.03%
WWTP	12/08/23	1,120,353	99.92%	98.74%	95.78%	92.16%	79.36%
WWTP	12/13/23	2,235,736	99.38%	97.67%	92.54%	87.64%	77.03%
WWTP	12/18/23	293,270	99.55%	97.94%	90.64%	82.13%	65.87%
WWTP	12/27/23	646,889	99.61%	97.69%	92.49%	86.53%	73.49%
WWTP	12/29/23	720,087	99.85%	99.04%	93.82%	89.63%	79.23%
WWTP	01/19/24	5,130,158	98.98%	98.80%	98.49%	98.30%	97.56%
WWTP	01/24/24	4,330,417	94.72%	90.08%	85.97%	83.04%	77.31%
WWTP	02/02/24	1,115,461	94.11%	84.85%	81.84%	80.61%	79.83%
WWTP	02/07/24	1,730,329	95.24%	88.41%	84.86%	82.86%	81.37%
WWTP	02/09/24	1,986,148	96.47%	90.24%	86.51%	84.97%	83.58%
WWTP	02/14/24	1,019,912	95.22%	88.32%	84.26%	82.91%	80.77%
WWTP	02/21/24	908,527	96.35%	92.84%	85.39%	80.23%	70.84%
WWTP	02/23/24	548,824	96.56%	88.01%	79.86%	71.82%	61.03%
WWTP	03/06/24	474,331	91.38%	85.42%	76.79%	70.62%	61.38%
WWTP	03/13/24	1,242,336	96.13%	90.74%	82.89%	74.87%	62.75%
WWTP	03/15/24	1,077,025	93.52%	86.90%	82.54%	78.91%	70.66%
WWTP	03/29/24	412,648	96.74%	88.92%	78.45%	70.76%	59.15%
WWTP	04/03/24	222,822	99.83%	96.29%	88.49%	81.84%	70.60%
WWTP	04/10/24	636,614	97.66%	93.15%	87.39%	82.51%	76.00%
WWTP	04/26/24	791,458	98.54%	96.88%	93.66%	88.93%	78.32%
WWTP	05/03/24	558,456	94.94%	82.83%	76.17%	70.81%	61.67%
WWTP	05/15/24	516,210	98.11%	94.61%	89.63%	82.90%	71.21%
WWTP	05/17/24	1,283,941	98.82%	96.55%	91.75%	88.27%	76.61%
WWTP	05/31/24	2,335,519	99.16%	98.16%	96.51%	94.76%	88.93%
WWTP	06/05/24	1,406,491	99.17%	98.15%	96.73%	94.36%	87.13%
WWTP	06/26/24	979,719	93.65%	83.34%	79.09%	77.00%	73.14%

Supplementary Table S2. Whole genome sequencing (WGS) metrics for the Airport manhole samples. For quality control, samples were required to achieve ≥70% genome coverage at 50x depth or greater.

Location	Date	Reads	Coveredby1x	Coveredby10x	Coveredby30x	Coveredby50x	Coveredby100x
Airport	11/10/23	664,899	99.51%	96.53%	88.96%	82.97%	71.90%
Airport	11/14/23	1,012,989	99.71%	98.62%	96.88%	94.30%	90.78%
Airport	11/21/23	1,021,525	99.89%	99.86%	99.75%	99.64%	97.38%
Airport	11/25/23	702,646	99.90%	99.51%	97.44%	94.13%	86.92%
Airport	12/01/23	583,721	99.85%	99.25%	97.87%	96.50%	92.62%
Airport	12/15/23	1,496,661	99.89%	99.53%	99.33%	98.10%	95.85%
Airport	12/19/23	284,578	99.23%	98.74%	94.70%	88.67%	79.25%
Airport	12/22/23	1,318,036	99.89%	99.85%	99.84%	99.39%	98.27%
Airport	01/03/24	1,028,168	99.89%	99.85%	99.85%	99.77%	98.44%
Airport	01/17/24	7,894,706	98.60%	96.13%	91.28%	88.86%	87.09%
Airport	01/19/24	7,961,586	98.87%	97.70%	94.16%	92.06%	90.30%
Airport	01/23/24	237,743	85.30%	79.31%	76.16%	73.38%	64.36%
Airport	01/26/24	1,864,617	100.00%	99.95%	99.93%	99.84%	99.38%
Airport	01/31/24	5,672,567	97.59%	93.40%	88.81%	86.97%	84.73%
Airport	02/01/24	506,158	84.85%	78.09%	74.21%	72.83%	70.37%
Airport	02/09/24	2,445,720	97.70%	92.56%	89.00%	87.51%	86.07%
Airport	02/11/24	1,429,556	95.88%	88.54%	85.12%	84.17%	82.06%
Airport	02/12/24	2,074,216	90.97%	83.05%	80.36%	79.75%	78.46%
Airport	02/13/24	1,665,954	93.99%	85.60%	81.87%	80.48%	79.39%
Airport	02/15/24	527,633	91.07%	83.62%	76.20%	72.30%	63.66%
Airport	02/20/24	795,433	95.97%	88.00%	80.78%	73.23%	65.33%
Airport	02/23/24	860,975	98.02%	94.29%	87.02%	83.35%	76.06%
Airport	02/27/24	4,495,267	90.82%	88.30%	86.11%	84.70%	80.74%
Airport	03/05/24	962,528	99.02%	97.55%	94.57%	90.95%	84.06%
Airport	03/12/24	591,525	97.61%	93.31%	84.95%	79.11%	67.89%
Airport	03/26/24	931,504	95.66%	86.24%	78.95%	73.26%	65.51%
Airport	04/02/24	879,009	98.84%	97.79%	95.15%	92.47%	86.68%
Airport	05/21/24	326,530	99.02%	96.71%	90.96%	84.62%	69.39%
Airport	05/24/24	275,707	97.87%	92.86%	82.18%	73.27%	58.65%
Airport	05/28/24	249,599	96.76%	91.51%	79.92%	71.31%	57.44%
Airport	06/04/24	992,832	98.23%	96.91%	93.02%	87.44%	78.95%
Airport	06/18/24	947,734	98.95%	95.58%	89.97%	85.34%	80.15%
Airport	06/21/24	1,036,996	97.51%	90.81%	86.82%	85.53%	82.84%
Airport	06/25/24	766,657	95.21%	85.97%	78.67%	75.72%	67.59%

Supplementary Table S3. Whole genome sequencing (WGS) metrics for the Bars manhole samples. For quality control, samples were required to achieve ≥70% genome coverage at 50x depth or greater.

Location	Date	Reads	Coveredby1x	Coveredby10x	Coveredby30x	Coveredby50x	Coveredby100x
Bars	11/12/23	1,323,023	100.00%	99.93%	99.77%	98.56%	93.17%
Bars	12/02/23	1,824,819	99.61%	98.90%	98.37%	97.82%	96.61%
Bars	12/03/23	387,473	99.80%	99.08%	95.79%	90.35%	79.43%
Bars	12/17/23	178,961	99.45%	95.88%	85.42%	76.55%	57.61%
Bars	12/23/23	757,644	99.60%	98.13%	96.12%	93.60%	85.34%
Bars	12/30/23	551,371	99.98%	99.96%	99.91%	99.64%	96.72%
Bars	12/31/23	1,142,482	99.85%	99.71%	98.96%	96.93%	92.84%
Bars	01/20/24	6,906,582	99.19%	98.25%	95.34%	92.57%	90.47%
Bars	01/27/24	2,460,555	92.55%	86.48%	82.45%	80.61%	79.20%
Bars	02/03/24	606,722	89.10%	80.54%	77.82%	77.06%	74.08%
Bars	02/11/24	2,437,269	89.05%	79.93%	75.22%	73.39%	70.91%
Bars	02/12/24	1,878,148	97.45%	90.32%	86.13%	84.65%	82.89%
Bars	02/17/24	1,142,062	98.87%	97.65%	95.01%	93.11%	86.62%
Bars	02/24/24	1,172,735	99.04%	97.80%	95.12%	92.93%	88.74%
Bars	03/16/24	1,681,409	99.00%	98.57%	97.38%	96.75%	92.83%
Bars	05/26/24	531,530	98.74%	96.96%	94.45%	89.95%	78.86%
Bars	05/27/24	501,217	98.33%	94.91%	89.23%	84.85%	75.14%
Bars	06/01/24	1,372,855	98.91%	98.00%	96.63%	95.52%	91.60%
Bars	06/22/24	1,499,968	97.53%	90.57%	86.43%	84.53%	83.04%
Bars	06/29/24	776,554	95.07%	86.11%	81.35%	79.28%	76.88%

Supplementary Table S4. Summary of observed PMMoV, SARS-CoV-2, and PMMoV-normalized SARS-CoV-2 concentrations as a function of location and method. "WWTP" and "Solids" concentrations are reported in units of gc/g, "Liquids" concentrations are reported in units of gc/L, and PMMoV-normalized SARS-CoV-2 concentrations are unitless but multiplied by 1 million for scaling purposes.

	AIRPORT								
	PMMoV			SARS-CoV-2			Normalized SARS-CoV-2[a]		
	WWTP	Solids	Liquids	WWTP	Solids	Liquids	WWTP	Solids	Liquids
Mean	5.90×10^{8}	4.48×10^{8}	1.48×10^{8}	2.31×10^{5}	4.60×10^{5}	9.41×10^{5}	4.62×10^{2}	1.14×10^{3}	7.71×10^{3}
St. Dev.	5.02×10^{8}	3.41×10^{8}	1.02×10^{8}	4.28×10^{5}	8.54×10^{5}	1.34×10^{6}	6.30×10^{2}	1.99×10^{3}	1.24×10^{4}
Min	9.97×10^{7}	3.79×10^{7}	2.82×10^{7}	1.15×10^{4}	ND	7.24×10^{4}	9.27×10^{0}	ND	7.21×10^{2}
25th	3.36×10^{8}	1.77×10^{8}	1.04×10^{8}	6.99×10^{4}	7.21×10^{4}	3.20×10^{5}	1.39×10^{2}	2.54×10^{2}	2.33×10^{3}
Median	4.70×10^{8}	3.87×10^{8}	1.31×10^{8}	1.43×10^{5}	1.60×10^{5}	5.02×10^{5}	2.67×10^{2}	5.70×10^{2}	4.50×10^{3}
75th	6.22×10^{8}	5.71×10^{8}	1.65×10^{8}	2.56×10^{5}	5.33×10^{5}	1.03×10^{6}	5.32×10^{2}	1.13×10^{3}	7.48×10^{3}
Max	3.44×10^{9}	1.69×10^{9}	8.35×10^{8}	4.29×10^{6}	6.25×10^{6}	8.99×10^{6}	5.56×10^{3}	1.38×10^{4}	8.51×10^{4}
	**BARS**								
	**PMMoV**			**SARS-CoV-2**			**Normalized SARS-CoV-2[a]**		
	**WWTP**	**Solids**	**Liquids**	**WWTP**	**Solids**	**Liquids**	**WWTP**	**Solids**	**Liquids**
Mean	5.90×10^{8}	7.69×10^{8}	1.11×10^{8}	2.31×10^{5}	6.82×10^{5}	8.67×10^{5}	4.62×10^{2}	1.32×10^{3}	7.37×10^{3}
St. Dev.	5.02×10^{8}	1.28×10^{9}	5.15×10^{7}	4.28×10^{5}	1.11×10^{6}	1.12×10^{6}	6.30×10^{2}	1.81×10^{3}	8.36×10^{3}
Min	9.97×10^{7}	1.09×10^{7}	3.55×10^{7}	1.15×10^{4}	ND	ND	9.27×10^{0}	ND	ND
25th	3.36×10^{8}	2.91×10^{8}	8.03×10^{7}	6.99×10^{4}	2.64×10^{4}	1.37×10^{5}	1.39×10^{2}	6.98×10^{1}	1.40×10^{3}
Median	4.70×10^{8}	3.66×10^{8}	1.01×10^{8}	1.43×10^{5}	1.30×10^{5}	5.09×10^{5}	2.67×10^{2}	3.88×10^{2}	4.87×10^{3}
75th	6.22×10^{8}	5.56×10^{8}	1.34×10^{8}	2.56×10^{5}	8.33×10^{5}	1.17×10^{6}	5.32×10^{2}	2.03×10^{3}	1.09×10^{4}
Max	3.44×10^{9}	7.01×10^{9}	3.08×10^{8}	4.29×10^{6}	4.35×10^{6}	5.29×10^{6}	5.56×10^{3}	6.25×10^{3}	3.87×10^{4}

[a] Normalized concentration ratio × 1 million

Supplementary Table S5. Spearman correlation coefficients and corresponding Fisher's exact p-values for weekly (Sunday-Saturday) mean longitudinal raw and PMMoV-normalized SARS-CoV-2 wastewater concentrations. Analyses were conducted by sampling location (i.e., wastewater treatment plant, or WWTP; airport; bars) and by sample fraction analyzed (i.e., solids-based, Verily Life Sciences; liquids-based, Biobot).

SARS-CoV-2 Concentration	Location 1	Fraction Analyzed	Location 2	Fraction Analyzed	N	Spearman coefficient	p-value
Raw	Airport	Solids	WWTP	Solids	37	0.49	0.0017
	Airport	Liquids	WWTP	Solids	37	0.67	<0.0001
	Bars	Solids	WWTP	Solids	31	0.59	0.0003
	Bars	Liquids	WWTP	Solids	29	0.76	<0.0001
	Airport	Solids	Bars	Solids	31	0.52	0.0026
	Airport	Solids	Bars	Liquids	29	0.64	0.0001
	Airport	Liquids	Bars	Liquids	29	0.67	<0.0001
	Airport	Liquids	Bars	Solids	31	0.58	0.0005
	Airport	Solids	Airport	Liquids	37	0.78	<0.0001
	Bars	Solids	Bars	Liquids	30	0.85	<0.0001
PMMoV-normalized	Airport	Solids	WWTP	Solids	37	0.45	0.0049
	Airport	Liquids	WWTP	Solids	37	0.51	0.0011
	Bars	Solids	WWTP	Solids	31	0.65	<0.0001
	Bars	Liquids	WWTP	Solids	29	0.59	0.0005
	Airport	Solids	Bars	Solids	31	0.49	0.0042
	Airport	Solids	Bars	Liquids	29	0.68	<0.0001
	Airport	Liquids	Bars	Liquids	29	0.70	<0.0001
	Airport	Liquids	Bars	Solids	31	0.51	0.0027
	Airport	Solids	Airport	Liquids	37	0.83	<0.0001
	Bars	Solids	Bars	Liquids	30	0.89	<0.0001
